# Management with colistin

**DOI:** 10.4103/0972-5229.74179

**Published:** 2010

**Authors:** Harkirat Singh

**Affiliations:** All India Institute of Medical Sciences, New Delhi, India

Dear Sir,

I read the article “Colistin and polymyxin B: A re-emergence”[1] with great interest. I heartily applaud the efforts of the authors to write this review article. However, I would like to add some more information in this regard.

Notably, *Proteus* spp., *Moraxella catarrhalis, Providencia* spp., *Serratia marcescens, Morganella morganii*, gram-negative cocci, and all gram-positive bacteria are resistant to colistin. Moreover, *Prevotella* and *Fusobacterium* spp. have variable sensitivity.[2] Hence, we should be careful using it against these bacteria.

Aerosolization of colistin into the airway can be complicated by bronchospasm, especially in patients with advanced lung disease and low baseline spirometry; bronchodilation prior to administration may be beneficial.[3] In addition, the prodrug colistimethate sodium should be reconstituted just before administration as nebulization so as to avoid excessive conversion to biologically active colistin, which can cause fatal airway or alveolar injury. In patients with pre-existing renal disease, dosage adjustments are required as impaired renal function may increase the risk for respiratory arrest.

Some more neurologic manifestations include psychosis, coma, convulsions, ptosis, diplopia, areflexia, dysphagia, and dysphonia.[4,5] Neuromuscular blockade is because of non-competitive blockade and thus it should not be used simultaneously with neuromuscular blocking agents. Capreomycin may also enhance this effect of colistin.

Prolonged use can cause fungal or bacterial superinfection, including *C. difficile*-associated diarrhea and pseudomembranous colitis; generally observed in more than 2 months postantibiotic treatment.
